# Assessment of pattern for consumption and awareness regarding energy drinks among medical students

**DOI:** 10.1186/2049-3258-71-31

**Published:** 2013-12-18

**Authors:** Hafiz Muhammad Aslam, Anum Mughal, Muhammad Muzzammil Edhi, Shafaq Saleem, Masood Hussain Rao, Anum Aftab, Maliha Hanif, Alina Ahmed, Agha Muhammad Hammad Khan

**Affiliations:** 1 Dow Medical College, Dow University Of Health Sciences, Karachi, Pakistan; 2 Liaquat National Medical College, Karachi, Pakistan; 3 Pakistan Medical Research Council, Karachi, Pakistan; 4 Jinnah Medical College, Karachi, Pakistan; 5 Sindh Medical College, Jinnah Sindh Medical University, Karachi, Pakistan

**Keywords:** Palpitation, Energy drinks, Behavior

## Abstract

**Background:**

Energy drink is a type of beverage which contains stimulant drugs chiefly caffeine and marketed as mental and physical stimulator. Coffee, tea, soft drinks and other caffeinated beverages are not considered as energy drinks. Purpose of our study was to evaluate the awareness of medical students regarding energy drinks and their pattern and reason of energy drinks consumption.

**Methods:**

This was a cross sectional and observational study conducted during the period of January – December 2012 at four Medical Colleges (Dow Medical College, Sindh Medical College, Jinnah Medical College and Liaquat National Medical College) of Karachi, Pakistan. Over all 900 M.B.B.S students were invited to participate after taking written consent but viable questionnaire was submitted by 866 students, estimated response rate of 96%. All data was entered and analyzed through SPSS version 19.

**Result:**

Out of 866 participants, majority were females 614 (70.9%) and only 252 (28.5%) were males, with a mean age of 21.43 ± 1.51 years. Energy drinks users were 350 (42.89%) and non users were 516 (59.58%). Only 102 (29.3%) users and 159 (30.7%) non users know the correct definition of Energy drinks. Regarding awareness, mostly user and non users thought that usage of energy drinks had been on rise due to its usefulness in reducing sleep hours [users193 (43.9%), nonusers 247 (56.1%) (p < 0.05)], for studying or completing major projects [users184 (45.0%), nonusers 225 (55.0%) (p < 0.05)] and for refreshment purposes [users179 (44.9%), nonusers 220 (55.1%) (p < 0.05)]**.** Two main reasons of not using energy drinks by non-users were “awareness from its side effects” 247 (47.8%) and “have no specific reason” 265 (51.3%). Most common side effects reported by users were fatigue 111 (31.7%) and weight gain 102 (29.4%).

**Conclusion:**

In sum, the fact that despite serious side effects of weight gaining and fatigue, practice of consuming energy drinks is highly prevalent among medical students, particularly because they are ever ready to boost their energy level and reduce sleep hours due to stress of exams and projects. This warrants the creation of continued public health awareness about the appropriate use of caffeinated beverages, their potential benefits, side effects and correction of wrong perceptions.

## Background

Energy drinks are caffeinated beverages first appeared in Europe and Asia in the 1960s but did not become popular until the most widely known brand, Red Bull, was released in Austria in 1987; hitting the US market in 1997. By 2006, there were over 500 brands of energy drinks around the world, with sales exceeding $500 million per annum in the US [1].

An energy drink is a type of beverage containing stimulant drugs, chiefly caffeine, which is marketed as mental and physical stimulator. Coffee, tea and other naturally caffeinated beverages are usually not considered as energy drinks. Soft drinks such as cola, may contain caffeine, but did not include in energy drinks [2]. Prevalence of energy drinks use among adolescent and young adults to be 30-50% [3]. Studies have also demonstrated its high consumption in young athletics [4].

Energy drinks generally contain methylxanthines (including caffeine), taurine, glucuronolactone, B Vitamins, and herbs. Other commonly used ingredients are carbonated water, guarana, yerba mate, açaí, and taurine. Probably they are responsible for the possible medical side effects of energy drinks [5]. Energy drinks are consumed by younger population for different reasons particularly for studying, partying, driving, for energy boost and wakefulness [6]. There is a positive association between energy drink consumption and improved psychomotor and mental performance because of the interaction of its various constituents [7]. In conjunction with its positive effects, there are reviews on the negative effects of energy drinks as well [8]. According to a study, energy drinks have shown its great effect on cardiovascular system in terms of modulating the heart rates and blood pressure [9]. It was also proven that its consumers have experienced neuropsychosis as caffeine induces psychosis in those without a previously diagnosed psychotic disorder [10]. Frequency of energy drink consumption was positively associated with “problem behaviors” exhibited as sexually risky behaviors, marijuana use, fighting, and failure to use seat belts [11]. It was also consider that genetic factors and pharmacological intolerance responsible for individual’s vulnerability to caffeine related disorders including caffeine intoxication, dependence, and withdrawal [12]. It also has erosion characteristics of dental enamel [13].

### Objectives

In Pakistan very little concentration has given on energy drinks and there is not much data available regarding the awareness, prevalence and side effects of consuming energy drinks. Whatever is available is severely lacking in quality and focuses on the percentage of side effects only, without fully covering the basic reasons and pattern of taking energy drinks**.** Therefore this study was planned to find out the awareness of medical students about the potential hazards of energy drink, their patterns and reason for consumption along with the knowledge of exact definition of energy drinks.

## Methods

### Data

A cross sectional and observational study was conducted during the period of January- December 2012. It was a multi-institutional study and all participants were students of different years of M.B.B.S studying in four large Medical Colleges of Karachi, i.e. Dow Medical College, Sindh Medical College, Liaquat National Medical College and Jinnah Medical College. Convenient sampling technique was used to collect the data. Approximately 900 students were contacted to participate in the study but viable questionnaires were submitted by 866 students, estimated response rate of 96%. Students of all other programs and qualification were excluded.

### Measures

For study seven trained researchers were included. Three students were from Dow Medical College, two from Sindh Medical College, one from Liaquat national medical college and one from Jinnah medical college. Initially questionnaire was field tested among 10 randomly chosen students who were in a public location on campus. The questionnaire took approximately two minutes to complete and modifications to the questionnaire were not necessary based on the field test responses. Researchers before giving questionnaire to anybody ensured that whom they approach were student at the university and that student had not previously completed the questionnaire.

### Ethical considerations

Study was initiated after taking approval from Institutional Review Board of Dow University of health Sciences. Prior written consent was taken from each student and they were also informed regarding study protocol. Those who were willing to participate anonymously completed the questionnaire.

### Questionnaire

Based on our objective, we made 16 variables questionnaire. Study instrument comprised of two sections. Section 1, which comprised of twelve questions (Q1-Q12), was concerned with the awareness of energy drinks usage.

#### ***Section I***

Q1 assessed demographic information (name, age, gender, name of medical college, year of study). Q2 was a questions with definition in which respondents were asked to choose energy drinks from various examples (coffee, tea, Pepsi, cola). In Q3, addiction behaviors were asked along with addiction of energy drinks.

Q4-Q9 assessed awareness among users and non users. In Q4 it was asked why people use energy drinks, Q5 was about the knowledge of withdrawal effects of energy drinks. In Q6 it was asked from respondents that energy drinks manufacturers claim many things, how much they agreed with manufacturers. Q7 assessed the awareness regarding side effects of energy drinks. Q8 had two sections “a” and “b”, in “a” section it was asked from participants that did they search about energy drinks in their life time, if participant give answer “yes” then they were instructed to give answer of section “b” which was about medium of searching, but if respondent marked answer “no” then it was instructed to skip section “b” and give answer of Q9. In Q9 it was inquire that according to their perspectives energy drinks is a fashion of upper class or not.

Q10 was a screening question, used to identify energy drink users, if participants answered “yes” then they were instructed to leave Q11 and fill rest of the Questionnaire, if respondent answer were “no” then they were instructed to give answer of question 11 only and return back the questionnaire to research assistant.

Q11 was only for non users in which reason of not drinking energy drinks was asked.

#### ***Section II***

From Q12-Q17, five questions were only for energy drinks users. Q12 was about the purpose for taking energy drinks, Q13 and Q14 were about withdrawal effects and time period of starting withdrawal effects respectively. Q15 and Q16 assessed pattern of consuming energy drinks, while Q17 was regarding side effects faced by users.

### Analysis

All the data was entered and analyzed through SPSS (Statistical Package for the Social Sciences) version 19. Frequency and percentage were calculated for categorical data and mean and standard deviation for continuous data. P values were calculated to determine the significance of association between users and non-users, and were based on the Chi-square test. Threshold of significance was set at <0.05. From table 1-3 percentages were calculated by making the 100% horizontally. For the calculation of percentage, divide the amount of users or non users with the total number of of users and non users in that particular row.

## Result

### Background information of study participants

According to the methodology of the study, 866 students were participated in the study. They belonged to different years of MBBS i.e. 100 (11.6%) from 1^st^ year, 259 (29.9%) from 2^nd^ year, 272 (31.4%) from 3^rd^ year, 218 (25.3%) from 4^th^ year and only 16 (1.8%) from final year. Study proportion comprised mostly of females 614 (70.9%), and males were only 252 (28.5%). Respondents were between ages of 18–25 years with a mean age of 21.43 ± 1.51 years. Students were from two government colleges i.e. Dow Medical college 210 (24.3%) and Sindh Medical College 207 (23.9%), and from two private medical colleges i.e. Liaquat National Medical College 251 (29.0%) and Jinnah Medical College 198 (22.8%) (Table 1).

**Table 1:** Socio-demographic characteristics of medical students

**Serial number**	**Variables**	**Energy drinks user N = 350**	**Energy drinks non-users N = 516**	**P value**
**1.**	Gender			
	1. Male	122 (48.8%)	130 (51.6%)	**0.002**
	2. Female	228 (37.1%)	386 (62.9%)	
**2.**	Name of colleges			
	A: Dow Medical College	89 (42.4%)	121 (57.6%)	**<0.01**
	B: Sindh Medical College	60 (29.0%)	147 (71.0%)	
	C: Liaquat National Medical College	105 (41.8%)	146 (58.2%)	
	D: Jinnah Medical College	96 (48.5%)	102 (51.5%)	
**3.**	Year of study			
	1st year	49 (49.0%)	51 (51.0%)	0.189
	2nd year	110 (42.5%)	149 (57.5%)	
	3rd year	108 (39.7%)	164 (60.3%)	
	4th year	77 (35.2%)	142 (64.8%)	
	5th year	6 (37.5%)	10 (62.5%)	

Medical students study of Dow, Sindh, Jinnah and Liaquat National Medical College, Karachi Pakistan 2012.

Threshold of significance is <0.05.

All significant values were in bold letters.

### Awareness and knowledge regarding energy drinks

Regarding knowledge of proper definition of energy drinks, 261 (30.1%). Knew the exact definition of energy drinks out of which 102 belong to users and 159 from non-users, while majority (69.9%) of participants didn’t know the exact definition of energy drinks. Most participants reported that they were also addicted to tea (p = <0.01). Regarding awareness of Energy drinks consumption’s patterns, mostly users and non users thought that it was taken for its significance in promoting wakefulness [users193 (43.9%), nonusers 247 (56.1%) (p = <0.05)], for studying or completing major projects [users184 (45.0%), nonusers225 (55.0%) (p < 0.05)], and for refreshment purpose [users179 (44.9%), nonusers 220 (55.1%) (p < 0.05)] (Table 2).

**Table 2:** Awareness regarding usage of energy drinks

**Serial number**	**Variables**	**Energy drinks user N = 350**	**Energy drinks non-users N = 516**	**P value**
1	Select energy drinks from the following?			
	Tea/coffee	155 (40.8%)	225 (59.2%)	
	red bull/lucozade/blue jeans	78 (42.9%)	104 (57.1%)	**0.015**
	string	21 (42.0%)	29 (58.0%)	
	burn/rox	2 (33.3%)	4 (66.7%)	
	lucozade/blue jeans	1 (4.2%)	23 (95.8%)	
	Pepsi/coke	41 (36.9%)	70 (63.1%)	
	All of these	52 (46.0%)	61 (54.0%)	
2.	Are you using any other substance of addiction?			
	Tea	106 (42.1%)	146 (57.9%)	
	Coffee	47 (57.3%)	35 (42.7%)	**<0.01**
	Cola drinks	31 (50.0%)	31 (50.0%)	
	Naswar	9 (64.3%)	5 (35.7%)	
	Cigarette	15 (60.0%)	10 (40.0%)	
	Heroin/charas	12 (54.5%)	10 (45.5%)	
	Alcohol	2 (8.7%)	21 (91.3%)	
	I don’t use any	128 (33.2%)	258 (66.8%)	
**3.**	*What is the most common adverse/withdrawal effect of energy drinks? (Multiple choice)			
	a. Fatigue	125 (47.0%)	141 (53.0%)	
	b. Dehydration	30 (34.5%)	57 (65.5%)	
	c. Increase heart rate	48 (26.5%)	133 (73.5%)	**0.05**
	d. Increase blood pressure	51 (39.8%)	77 (60.2%)	
	e. Tremors	11 (15.5%)	60 (84.5%)	
	f. Muscle stiffness and aches	23 (43.4%)	30 (56.6%)	
	g. Vomiting ,nausea and abdominal pain	36 (29.0%)	88 (71.0%)	
	h. Insomnia	57 (37.3%)	96 (62.7%)	
	i. Inability to focus	91 (47.2%)	102 (52.8%)	
	j. Headache	46 (39.7%)	70 (60.3%)	
	k. No adverse or with drawl effect it is just rumors	71 (54.6%)	59 (45.4%)	
	l. I don’t know	99 (39.9%)	149 (60.1%)	
**4.**	*Why people use energy drinks? (multiple choice)			
	a. Promote wakefulness	193 (43.9%)	247 (56.1%)	
	b. Need more energy	104 (31.0%)	231 (69.0%)	
	c. Studying/completing major project	184 (45.0%)	225 (55.0%)	**0.05**
	d. Driving for long time	37 (45.1%)	45 (54.9%)	
	e. While partying	100 (50.5%)	98 (49.5%)	
	f. Weight loss	11 (34.4%)	21 (65.6%)	
	g. Athletic performance	34 (29.3%)	82 (70.7%)	
	h. Refreshment/taste	179 (44.9%)	220 (55.1%)	
	i. Attract from advertisement	43 (35.0%)	80 (65.0%)	
	j. Relief stress	111 (42.7%)	149 (57.3%)	
**5.**	*Manufacturer claims that energy drinks do many things, for how many thing you agree? (multiple choice)			
	a. Physical endurance reaction	67 (34.4%)	128 (65.6%)	**0.05**
	b. Concentration and memory recall	142 (50.0%)	142 (50.0%)	
	c. Decrease sleep	194 (43.8%)	249 (56.2%)	
	d. Increase the ability of decision making	48 (36.4%)	84 (63.6%)	
	e. give you extra amount of energy	162 (45.0%)	198 (55.0%)	
	f. helps in athletic and academic performance	72 (44.2%)	91 (55.8%)	
	g. don’t agree with any one of them	39 (24.1%)	123 (75.9%)	
	h. I don’t know	54 (35.5%)	98 (64.5%)	

Medical students study of Dow, Sindh, Jinnah and Liaquat National Medical College, Karachi Pakistan 2012.

Threshold of significance is <0.05.

All significant values were in bold letters.

“*” indicates multiple choice questions.

In regard to awareness about withdrawal effect, mostly users and non user considered “fatigue” [users125 (47.0%), nonusers 141 (53.0%) (p < 0.05)] was the most common withdrawal effect while other side effect indicated by users was “tachycardia” 48 (26.5%), and by non users was “inability to focus” 102 (52.8%) (p < 0.05) (Table 2).

Regarding manufacturers claim, both users and non users were agreed to the fact that energy drinks decrease sleep [users 194 (43.8%), nonusers 249 (56.2%) (p < 0.05)], and boost extra amount of energy for work [users162 (45.0%), nonusers 198 (55.0%) (p < 0.05)] (Table 2).

About its health effects, both users and non users was on the same table and claimed that it was injurious for human health [users189 (34.6%), nonusers 357 (65.4%) (p = <0.01)] (Table 3).

**Table 3:** Awareness regarding energy drink’s usage

**Serial number**	**Variables**	**Energy drinks user N = 350**	**Energy drinks non-users N = 516**	**P value**
**1.**	Do you think energy drink consumption is good for human health?			
	a. Yes	97 (60.6%)	63 (39.4%)	**<0.01**
	b. No	189 (34.6%)	357 (65.4%)	
	c. I don’t know	64 (40.0%)	96 (60.0%)	
**2**	Do you think energy drinks cause palpitations, hypertension and heart problems?			
	Yes	188 (36.6%)	325 (63.4%)	**<0.01**
	No	87 (59.7%)	58 (40.3%)	
	I don’t know	75 (36.0%)	133 (63.6%)	
**3**	Do you think energy drinks cause neuropsychosis?			
	Yes	120 (40.8%)	174 (59.2%)	0.448
	No	73 (36.7%)	126 (63.3%)	
	I don’t know	157 (42.1%)	216 (57.0%)	
4	Do you think energy drinks cause weight gain?			
	Yes	210 (42.3%)	287 (57.7%)	
	No	74 (40.9%)	107 (59.1%)	0.233
	I don’t know	66 (35.1%)	122 (64.9%)	
5	Do you think energy drinks cause dental caries?			
	Yes	239 (43.0%)	317 (57.0%)	0.098
	No	55 (34.2%)	106 (65.8%)	
	I don’t know	56 (37.6%)	93 (62.4%)	
6	Do you think consuming energy drinks consuming is the symbol of fashion or high class status?			
	Yes	189 (42.9%)	252 (57.1%)	0.147
	No	161 (38.1.0%)	264 (62.11%)	
7	Had you ever searched about energy drinks?			
	Yes	141 (61.0%)	90 (39.0%)	**<0.01**
	No	209 (32.9%)	426 (67.1%)	
	If yes from where:			
8	Reading in any journal/book	54 (61.3%)	34 (38.6%)	**<0.01**
	From a friend or relative	18 (56.3%)	14 (43.8%)	
	Watching on a T.V.	65 (76.5%)	20 (23.5%)	
	From newspaper	4 (15.4%)	22 (84.6%)	

Medical students study of Dow, Sindh, Jinnah and Liaquat National Medical College, Karachi Pakistan 2012.

Threshold of significance is <0.05.

All significant values were in bold letters.

Knowledge about side effects of energy drinks was also assessed. According to the outcomes, majority of participants thought that main side effects of energy drinks were gaining weight [users210 (42.3%), nonusers 287 (57.7%) (p = 0.233)] and development of dental caries [users239 (43.0%), nonusers 317 (57.0%) (p = 0.098)]. Significant number of users considered energy drinks as one of the culprit of rising cardiovascular [188 (36.6%)] and neuropsychotic [120 (40.8%)] problems in society (Table 3).

Comparatively users 141 (61.0%) (p = <0.01) had searched more about energy drinks (p = <0.00) and the most common medium they used for searching was Television 65 (76.5%), while mostly non-users were became familiar with energy drinks by the help of medical journals and books 36 (39.1%) (p = <0.00) (Table 3).

### Non-users

**Figure 1:**
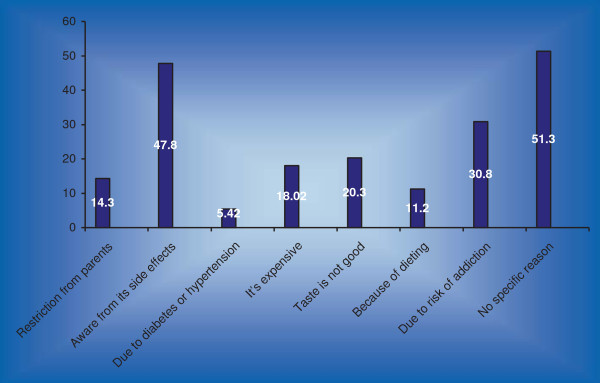
**Reasons of non-users of not using energy drinks.** Medical students study of Dow, Sindh, Jinnah and Liaquat National Medical College, Karachi Pakistan 2012.

Majority of non-users reported that there was no specific reason 265 (51.3%) of not drinking energy drinks while awareness from side effects 247 (47.8%) was also one of the main reason for not drinking energy drinks (Figure 1).

### Pattern of consumption and side effects face by energy drinks users

**Figure 2:**
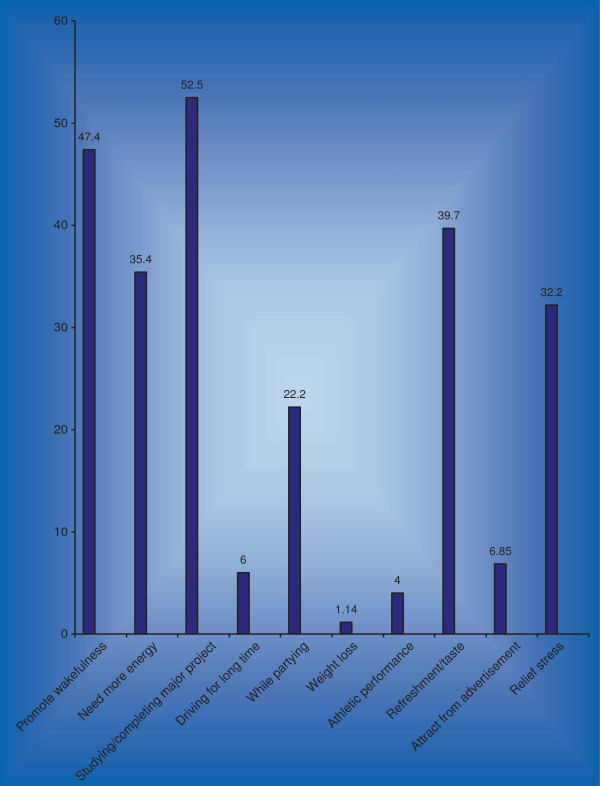
**Reasons of users for consuming Energy drinks.** Medical students study of Dow, Sindh, Jinnah and Liaquat National Medical College, Karachi Pakistan 2012.

Prevalence of users of energy drinks among medical student is 350 (49.2%). Most of the participants used energy drinks for the purpose of promoting wakefulness 166 (47.4%), completing their study projects 184 (52.5%) and for boosting energy levels 124 (35.4%) (Figure 2).

Commonest withdrawal effect faced by most users were “fatigue” 111 (31.7%) and “inability to focus” 61 (17.42%) which **c**ame within 12–24 hours 63 (18%). Mostly consume energy drinks during studying 188 (53.7%) and stress 116 (33.1%). Majority of users experienced weight gain 102 (29.14%), after taking energy drinks. Frequency of taking energy drinks by most users in past 12 months were 1 cane [124 (35.4%)] for daily 142 (40.57%) (Table 4).

**Table 4:** Pattern of energy drinks usage

**Serial number**	**Variables**	**Energy drinks users**
1	*What is the most common adverse/withdrawal effect of energy drinks you experience? (multiple choice)	
	Fatigue	111 (31.7%)
	Dehydration	49 (14%)
	Increase heart rate	55 (15.7%)
	Increase blood pressure	33 (9.42%)
	Tremors	29 (8.28%)
	Muscle stiffness and aches	14 (4%)
	Vomiting ,nausea and abdominal pain	37 (10.57%)
	Insomnia	42 (12%)
	Inability to focus	61 (17.42%)
	I don’t know	60 (17.14%)
	No adverse and with drawl effect come ,according to my experience, they are just rumors	106 (30.28%)
	Headache	39 (11.14%)
2a	In past 12 month how often you use it	
	Daily	142 (40.57%)
	Weekly	107 (30.5%)
	Monthly	101 (28.8%)
2b	How many canes or bottle of energy drinks did you use daily, weekly or monthly in Past 12 months?	
	1 cane	124 (35.4%)
	2 cane	70 (20%)
	3 cane	27 (7.71%)
	4 cane	29 (8.28%)
	1 bottle	28 (8%)
	2 bottle	32 (9.14%)
	3 bottle	13 (3.71%)
	4 bottle	27 (7.71%)
3	Withdrawal/adverse effects are mostly seen after_____?	
	Immediately	44 (12.57%)
	Before 12 hours	34 (9.71%)
	12–24 hours	63 (18%)
	After 3 days	16 (4.57%)
	After a week	20 (5.71%)
	No withdrawal effects	114 (32.57%)
	No specific time	59 (16.85%)
4	What do you experience after taking energy drinks?	
	Experience weight gain	102 (29.14%)
	Experience palpitation	58 (16.57%)
	experience tachycardia and hypertension	66 (18.857%)
	Experience dental caries	66 (18.57%)
	Experience neuropshychosis	58 (16.57%)
5	*What is your pattern of drinking energy drinks? (multiple choice)	
	During studying	188 (53.7%)
	During partying	72 (20.5%)
	During taking food	65 (18.5%)
	During driving	42 (12%)
	During free time	77 (22%)
	Before any sports	15 (4.28%)
	During stress	116 (33.1%)

Medical students study of Dow, Sindh, Jinnah and Liaquat National Medical College, Karachi Pakistan 2012.

“*” indicates multiple choice questions.

## Discussion

Energy drink consumption has been continued to gain popularity since its inception in Australia in 1987 and in United States in 1997. From the day of launching till today its market has grown rapidly with nearly 500 new brands launched worldwide in 2006 and 200 new brands launched in the United States in 12 month period in 2007 [6,8,12].

Energy drinks have attained more prominence in young adult market. They are designed to enhance alertness or provide short term memory boost and are readily available at college campuses and recreational hot spots.

Results from present study indicate a greater prevalence of energy drinks consumption among males. Findings of our study corroborate those of similar studies in which it was found that male consumed more servings of energy drinks [14,15]. Reason behind the findings can be advertisement of energy drinks which primarily targets adult male; furthermore males have more urge to achieve success as compared to females. Most men are competitive, accept challenges and tend to be stimulated by situation involving task or role accomplishment and assume risk, compared with females. This could be the possible reason for consuming energy drinks more often and in higher quantities than female [4,16].

Energy drinks are promoted for their stimulatory effect and claim to offer a variety of benefits including physical endurance reaction, concentration and memory recall, decrease sleep, increase ability of decision making give extra amount of energy and promote athletic performance. Majority of these claim however remained substantiated. The most consistent result to emerge was that intake of Energy drinks can increase long term physical endurance; improve cognitive ability and energy output. Our findings were consistent with past study [12]. Primary ingredient in energy drinks that has a cognitive function is the caffeine. Low doses of caffeine (12.5-50 mg) has been found to improve cognitive performance and mood and 200 mg doses have been found to improve cognitive task, speed, accuracy, increase alertness and the amount of caffeine provided in energy drinks an easily far exceed the amount necessary to promote cognitive function [6].

A common reason given by 2/3rd respondents regarding why they drink energy drinks was to reduce sleep and boost energy level for study and completing projects. This may be due the reason that caffeine increase cortisol secretion by stimulating Central Nervous System and causes sensitization of a specific subset of cannabinoid receptor in the striatum, consistent with the psychoactive properties of the compound. This explains why enhanced relaxation and sense of well being occurs during use of caffeine in stressful event [17].

Energy drinks usage has now become wide spread among college students, particularly who want to meet both cognitive and physical performance demand [4,14]. High intake of energy drinks, particularly brand that contain high quantity of caffeine can result in the slow downing the rate at which nutrient is absorbed into blood stream; it also slow downs the rate of fluid absorption or dehydration during an exercise. Excessive caffeine provides a blast of energy enabling the person to feel good initially but when energy is burn up in 30–40 minutes, there is a sugar crash [4].

Findings of our study is also consistent with past study which shows that person who consumed energy drinks reported less sleepiness and increased alertness [18]. Approximately 15.3% person claim dehydrating effect of energy drinks on their body which was consistent with past studies [4,19]. It may be due to the fact that there were serious consequences when a person substitute energy drinks for water during strenuous physical activity, this is because caffeine act as diuretic agent and it removes extra fluid from the body therefore if a person consume it while sweating, and it will result in severe dehydration [4].

In our setup prevalence of insomnia due to energy drinks was around 17% which was much less than a study occur in Thailand [20]. Most subject who reported recreational use experience symptoms such as palpitation, tremors, seizures, inability to focus, accelerated heart rate and gastrointestinal upset, the same as reported in past studies [21-23]. Elevation of blood pressure and heart rate was may be due to the pressor effect of caffeine which cause peripheral vasoconstriction rather than enhancement of cardiac output [24].

Energy drinks target market is different than in some of the other beverage industries. When they were first being sold in United States, athletes were primary consumers. But now marketing has been expanded beyond that of simply athletes. Although everyone is susceptible to the fatigue of the super-charged, over-worked lifestyle but young people are especially vulnerable to persistent exhaustion and insufficient energy. This group of people, more specifically male teenagers and people in their 20s and 30s, are also most likely to believe in the veracity of the energy drinks’ claims. As a result, the majority of energy drinks are developed for and advertised to this younger generation. Same results were found in our study as mostly users of energy drinks start using them by watching its advertisement on television.

Symptoms of caffeine withdrawal including fatigue, insomnia, muscle aches, irritability, and depression begin in 12–24 hours after the last dose of Energy drinks; our findings were consistent with past study [12].

### Strength and limitations

There is a distinct shortage of large scale generalized studies that map out prevalence as well as demographically specific consumption pattern and this present analysis offer preliminary data regarding prevalence, pattern of consumption and knowledge towards Energy drinks. Strength of our study lies in assessing awareness regarding energy drinks and its pattern of consumption among medical students. All attempts were made to ensure that the data collected was reliable and the methods were reproducible. Our study was also not free from limitations. Most important limitation for our study was that it was conducted in just four medical colleges. Although, these medical colleges consist of a heterogeneous population coming from different backgrounds and socioeconomic conditions, they cannot be used to predict the overall situation in the country. Furthermore, convenient sampling was employed, which may have led to selection bias, and hence was not truly representative of the population under study. Another limitation that could have affected the outcome of our study was the possibility of recall bias with regard to the pattern and side effects of energy drinks.

### Suggestion for future studies

Suggestions for further studies include assessing whether students have any knowledge of the active ingredients in energy drinks and whether they have the right information about the potential positive and negative effects of each ingredient which was used in energy drinks.

### Recommendations

• Energy drinks contain as much caffeine as a cup of coffee. Its usage must be done in limit because too much caffeine may cause nervousness, irritability, sleeplessness, and, occasionally, rapid heartbeat. It could be used for occasional use only.

• Never intended for use as a substitute for sleep or gaining extra energy.

• If fatigue or drowsiness persists or continues to recur, consult a physician immediately. It is also suggested that due to its side effects it should not be given to children under 12 years of age.

• Parents and peers should play a valuable role in monitoring risk for caffeine related consequences among energy drink users.

• Young adults should be educated about the risks of drinking energy drinks.

• Health care provider must inform the public on the potential health hazards related to excessive intake of caffeine-containing beverages by Adults, children and adolescents.

## Conclusions

More research and increased public awareness is needed to bring about a greater understanding of their effects. Given the positive and negative effects of energy drinks referenced above, there is no doubt that these beverages may provide consumers with temporary benefits, including increased cognitive performance, increased or maintained mood, more physical energy, and promotion of wakefulness. However, while these beverages may provide a quick fix to temporary difficulties faced by the consumers, the prolonged and continued use of these drinks may affect the health of an individual. In our study mostly medical students were aware of hazardousness of energy drinks but ratio has not much large and awareness has still needed.

## Competing interest

The authors declared that they had no competing interests.

## Authors’ contribution

HMA AM MME SS did analyzing and manuscript drafting. MHR did review critically and also supervise. AF MF AA AMHK did data collection and checks the manuscript. All authors check and approve the final version.
